# Evaluation of processing mechanism in Astragali Radix by low-field nuclear magnetic resonance and magnetic resonance imaging

**DOI:** 10.1371/journal.pone.0265383

**Published:** 2022-03-14

**Authors:** Jie Peng, Lifang Ye, Mengmei Wu, Menghua Wu, Zhiguo Ma, Hui Cao, Ying Zhang

**Affiliations:** 1 Research Center for TCM of Lingnan (Southern China), Jinan University, Guangzhou, P. R. China; 2 National Engineering Research Center for Modernization of Traditional Chinese Medicine Lingnan Resources Branch, Guangzhou, P. R. China; 3 Guangdong Key Laboratory of Traditional Chinese Medicine Information Technology, Guangzhou, P. R. China; Macau University of Science and Technology, MACAO

## Abstract

Astragali Radix (*Huangqi*) is an important herb medicine that is always processed into pieces for clinical use. Many operations need to be performed before use, among which drying of Astragali Radix (AR) pieces is a key step. Unfortunately, research on its drying mechanism is still limited. Low-field nuclear magnetic resonance (LF-NMR) and magnetic resonance imaging (MRI) techniques were applied to study the moisture state and distribution during drying. The content of bioactive components and texture changes were measured by HPLC and texture analyzer, respectively. The moisture content of the AR pieces decreased significantly during drying, and the time to reach the drying equilibrium were different at different temperatures. The time when at 70°C, 80°C, and 90°C reach complete drying are 180 min, 150 min and 120 min, respectively. 80°C was determined as the optimum drying temperature, and it was observed that the four flavonoids and astragaloside IV have some thermal stability in AR pieces. When dried at 80°C, although the total water content decreased, the free water content decreased from 99.38% to 15.49%, in contrast to the increase in bound water content from 0.62% to 84.51%. The texture parameters such as hardness changed to some extent, with the hardness rising most significantly from 686.23 g to 2656.67 g. Correlation analysis revealed some connection between moisture content and LF-NMR and texture analyzer parameters, but the springiness did not show a clear correlation with most parameters. This study shows that HPLC, LF-NMR, MRI, and texture analyzers provide a scientific basis for elucidating the drying principles of AR pieces. The method is useful and shows potential for extension and application; therefore, it can be easily extended to other natural herb medicines.

## Introduction

Processing, known as *Paozhi* in Chinese, is a unique Chinese pharmaceutical technique with a long history of facilitating the use of Chinese herbal medicines for specific clinical needs based on traditional Chinese medical theory [1]. Traditional Chinese medicine decoction pieces, known as *Yinpian* in China, are important processed product that is directly applicable for clinical use [2]. Except for a few that can be used when fresh, most *Yinpian* need to be kept dry before use [3]. The dried medicinal materials in production are removed from impurities, cleaned, moistened, cut and dried, and then different decoction pieces are made accordingly. Drying is the key step in removing the internal moisture of herbs for storage [4].

Astragali Radix (*Huangqi* in Chinese) originates from the dried root of *Astragalus membranaceus* (Fisch.) Bge. var. *mongholicus* (Bge.) Hsiao or *Astragalus membranaceus* (Fisch.) Bge., which has been used as traditional Chinese medicine (TCM) for more than 2,000 years [5]. Astragali Radix (AR) can tonify the middle Jiao and Qi, solidify the surface and cause water swelling, dispel sepsis and renew muscle [6]. Modern research shows that AR has many biological functions, such as vasodilation [7], antioxidant activity [8, 9], immunomodulation [10, 11], antiaging activity [12, 13], and antitumor activity [14]. The process of producing AR decoction pieces is to ’’remove impurities, separate size, clean, moisten, cut thick pieces and dry [15].’’ At present, research reports on the drying of AR decoction pieces are shallow and mainly use appearance, shape and other indicators to pursue an optimum drying process. There is a lack of research on the drying mechanism. Therefore, it is impossible to guarantee the quality of the pieces after the drying process and to optimize the process parameters. Meanwhile, there are many reports on the drying mechanisms of fruits, vegetables and seafood, such as strawberries [16], garlic [17], carrots [18] and abalone [19]. The aforementioned studies usually use a combination of multiple techniques to monitor physical property changes during drying. Low-field nuclear magnetic resonance (LF-NMR), magnetic resonance imaging (MRI) and texture analyzers have obvious advantages in detecting moisture migration, distribution and transformation and texture changes.

LF-NMR and MRI are powerful tools for analyzing the states and distributions of water in food matrices due to their fast analysis speed, sensitivity, and low cost. They are also noninvasive and nondestructive [20]. Moist herbal medicines contain a large amount of water, which ensures a strong signal in NMR relaxometry/NMR imaging, making them an ideal sample for these methods. The transverse relaxation time (T_2_) mainly provides information about the water state and water binding in the matrix. At the same time, MRI shows the spatial distribution of water and the changes during processing [21]. In addition, the texture analyzer is a versatile physical property detector. It has various test modes, such as compression, puncture, shear and tensile. Characterization of hardness, adhesion, elasticity, cohesion and other physical property parameters, the instrument is objective, sensitive, and accurate [22].

The aim of this study was to evaluate the drying characteristics of AR pieces at different drying temperatures and then to select the optimal drying temperature. Changes in the physicochemical properties of AR pieces during drying will also be monitored. The water state, migration, distribution and transformation of AR pieces dried at different times will be determined by LF-NMR and MRI technologies. The drying kinetic curves will elucidate the dynamic changes in moisture and mass during the drying process of AR pieces. HPLC and texture analyzers will be used to detect changes in chemical composition and texture during the drying process. The useful data obtained in this study were subjected to Pearson correlation analysis to derive the relationship between the parameters and thus provide a reliable solution for further research. These findings will help to explain the mechanism of AR pieces preparation, providing a new idea and model for the study of traditional Chinese medicine preparation.

## Materials and methods

### Materials

Astragali Radix was collected in Min County (Gansu Province, China) in September 2020 and was identified as the dried root of *Astragalus membranaceus* (Fisch.) Bge var. *mongholicus* (Bge.) Hsiao by Dr. Ying Zhang at the Research Center for Traditional Chinese Medicine of Lingnan (Southern China) of Jinan University. The herbal specimens were divided into three parts and stored in the specimen cabinet of the Research Center for TCM of Lingnan (Southern China), Jinan University.

### Chemical and standards

HPLC-grade phosphoric acid was obtained from Shanghai Macklin Biochemical Co., Ltd. (Shanghai, China). HPLC-grade methanol and acetonitrile were purchased from Thermo Fisher Scientific (Fair Lawn, NJ, USA). Purified water for the chromatographic mobile phase was purchased from China Resources Yibao Beverage (Guangzhou, China). Other reagent solutions were of analytical grade (Guangdong Guanghua Sci-Tech Co., Ltd., Shantou, China).

The standard substances calycosin-7-*O*-*β*-*D*-glucopyranoside (batch number: RFS-M02001902019), ononin (batch number: RFS-M01301904002), calycosin (batch number: RFS-M02101903026), formononetin (batch number: RFS-C01811812016) and astragaloside IV (batch number: RFS-H01301907008) were purchased from Chengdu Herbpurify Co., Ltd. (Chengdu, China). Their exact structural formulae were determined by hydrogen and carbon spectroscopy, and their purity was more than 98% measured by liquid chromatography.

### Drying experiments with AR pieces

The raw AR pieces were homemade in the laboratory: 600 g of AR was well moistened in distilled water, and then the AR was cut into pieces of 2–4 mm in diameter. The above pieces were evenly divided into three parts, placed in a single layer on a drying tray and dried in an electric blast furnace (Tianjin Taisite Instrument Co., Ltd., 101-2AB, Tianjin, China) at temperatures of 70°C, 80°C, and 90°C. The thickness of the layer was approximately 4–8 mm. Samples were weighed every half hour to obtain the moisture content at different drying time until the weight was constant within a certain range. The drying process will stop when the weight difference between two adjacent time is within 1% of the total weight, which is considered to be a safe level for compliance with storage requirements.

### Moisture content, moisture ratio and drying rate

Three parameters of the drying characteristics, moisture content (MC), moisture ratio (MR) and drying rate (DR), were measured in this section.

#### Moisture content

First, the moisture contents (g/g, d. b.) of AR pieces were calculated as follows:

$$MC_{t}=\left(m_{t}-m_{e}\right)/m_{e}$$ (1)

where *MC*_*t*_ is the moisture content of dried AR pieces at time *t*, *m*_*t*_ is the mass of AR pieces at time *t*, and *m*_*e*_ is the mass of the dry base.

#### Moisture rate

The moisture ratio (MR) can be expressed according to Eq (2):

$$MR=\left(MC_{t}-MC_{e}\right)/(MC_{0}-MC_{e})$$ (2)

where *MC*_0_ and *MC*_*e*_ are the initial water content (g/g, d. b.) and the final equilibrium moisture content of AR pieces. *MC*_*e*_ can be assumed to be zero when compared with *MC*_0_.

#### Drying rate

The drying rate (DR) represents the reduction in moisture content per unit time, and which can be expressed by the following formula:

$$DR=\left(MC_{t}-MC_{t+\Delta t}\right)/\Delta t$$ (3)

where *MC*_*t*_ and *MC*_*t+Δt*_ are the moisture content (g/g, d. b.) at times *t* and *t+Δt*, respectively.

### Determination of bioactive ingredient content by HPLC

The AR pieces dried at different times were smashed and filtered through a 65 mesh sieve. The extracts were prepared according to the procedure described in the Chinese Pharmacopoeia (2020 edition) [15].

With some modifications of the previous study [23], HPLC-DAD analysis was performed on a COSMOSIL C18 column (5C18-MS-II packed column, 4.6 mm × 250 mm, 5 μm, code 38020–41). The mobile phase was prepared from 0.5% aqueous phosphoric acid solution (A) and acetonitrile (B). The column temperature was maintained at 35°C. The injection volume was 10 μL. Signal monitoring was performed at 250 nm for all the tested compounds. The gradient elution program for HPLC was as follows: 20–35% B in 0–5 min; 35–50% B in 5–15 min; 50–65% B in 15–30 min; and post run (5 min) at a flow rate of 1 mL/min.

On the basis of previous research [24], HPLC-ELSD analysis of astragaloside IV, acetonitrile (phase A) and water (phase B) were used as the mobile phases. The gradient elution was programmed as follows: 0–10 min, 15–40% A; 10–20 min, 40% A. The drift tube temperature was set at 110°C, and air was used as the gas with the flow rate of 3.0 mL/min.

### LF-NMR and MRI analysis

Relaxation (T_2_) analysis was carried out using a MesoMR23-060H-I nuclear magnetic resonance analyzer (Suzhou Niumag Analytical Instrument Co., Ltd.) at 21 MHz The equipment was equipped with a thermostat to control the temperature at 32 ± 0.01°C to ensure the accuracy of the experimental results. The magnetic field strength was 0.5 T permanent, the radio frequency diameter is 60 mm, with 90° (P1) and 180° (P2) pulse times of 10 μs and 19.04 μs, respectively, and P1 and P2 are supplemented at intervals of 1 ms. The samples were equilibrated to room temperature to measure the transverse relaxation time T_2_. The measurement conditions were as follows: NECH (number of echoes) = 8,000, SW (signal receiving bandwidth) = 100 kHz, TW (time waiting) = 5,000 ms, NS (number of scans) = 4, TE (time echo) = 0.30 MS. The relaxation signals were collected by NMR Analysis software (Suzhou Niumag Analytical Instrument Co., Ltd.) and Carr–Purcell–Meiboom–Gill (CPMG) pulse sequences. Perform inversion with 100,000 simultaneous iterative reconstructions to obtain the relaxation map of the samples.

MRI was performed with the same LF-NMR analyzer and spin-echo sequence. The sample was placed in the center of the radio frequency (RF) coil to collect the signal and obtain a T_2_-weighted image. Image processing software was used to draw and process MRI images and eliminate background noise. The main parameters were as follows: slice width = 2.0 mm, slice gap = 1.0 mm, slice number = 5, TE (time echo) = 20 ms, read size = 256, phase size = 192, TR (time repetition) = 1000 ms, flip angle = 90°, refocus flip angle = 180°, averages = 4, RG = 20 dB, PRG was high, FOV = 100 × 100 mm.

### Measurement of texture parameters

The texture profiles of the samples were determined only in the center part by a texture analyzer (CT3, Brookfield Engineering Laboratory Instrument Co., Ltd., Guangzhou, China). In the compressed model, the probe was TA9 (needle, 1.0 mm in diameter, 43 mm in length). The parameters were as follows: trigger point load = 5 g, load cell = 10,000 g, return speed = 1 mm/s, test speed = 1 mm/s, puncture depth = 6 mm, and data frequency = 50 points/sec. The texture data were automatically analyzed by texture loader software (Brookfield Engineering Laboratory Instrument Co., Ltd., Guangzhou, China). Typical textural variables measured on AR pieces were hardness, adhesion, springiness, and fracturability.

### Statistical analysis

All the original data obtained were first imported into an EXCEL spreadsheet and analyzed by Statistical Package for the Social Sciences software, version 24 (SPSS, Chicago, IL, USA). The significance of differences between means was determined by one-way analysis of variance (ANOVA) to determine the determination, multiple post comparison selection Duncan’s multiple range test. The statistical significance of differences was tested at the 5% probability level (p < 0.05). Graphs were produced using Origin 2018 64 Bit software (Origin Laboratory, Northampton, MA, USA) and GraphPad Prism v.8.0 software (San Diego, CA, USA). All the above experiments were performed in triplicate and the average values were used.

## Results

### Drying characteristics

**Fig 1:**
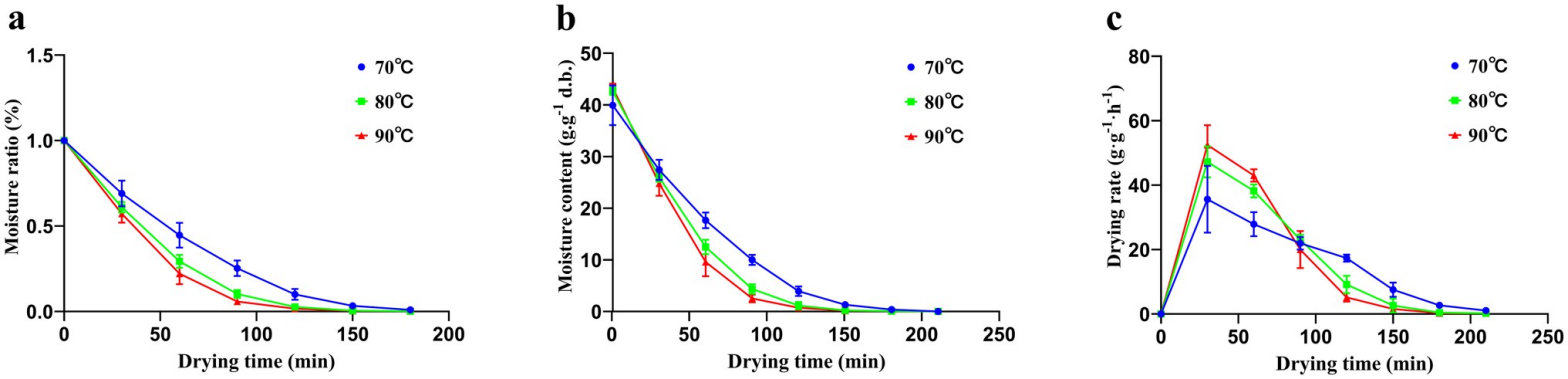
Drying characteristics of AR pieces. (a): Moisture ratio. (b): Moisture content. (c): Drying rate. Data are represented as the mean ± standard deviation of three independent experiments.

Combining previous studies and laboratory pre-experiments, we chose 70, 80 and 90°C for the experiments [25]. The drying characteristic curves for AR pieces during hot air drying at different temperatures are shown in Fig 1. The drying times required to reach the equilibrium moisture content for AR were 180, 150, and 120 min at 70°C, 80°C, and 90°C, respectively. Fig 1a shows that the higher the temperature was, the shorter the time required to reach the same moisture content. Compared with the drying time required at 70°C, those at 80°C and 90°C were 16.67% and 33.33% less, respectively. The moisture ratio (MR) curves decreased exponentially with drying time (Fig 1a). The curve with a higher steepness had a higher drying rate. As shown in Fig 1b, the 90°C drying curve showed the steepest slope, followed by the curves for 80°C and 70°C, which intuitively showed that the drying rate of 90°C was the fastest. Although the MR of the 90°C sample decreased faster than that of the 70°C sample, it was not significantly different from that of the 80°C sample. However, the 70°C sample required a longer time than the 90°C or 80°C samples to reach the final moisture content.

The curves for drying rate are shown in Fig 1c. When the drying time was 30 min, the drying efficiency at the three different temperatures all reached the maximum. Before 90 min, there was considerable water in the AR pieces, and a higher temperature caused more water loss per unit time. The drying rate curves were arranged from high to low temperatures. At 90 min, the drying rates of the three different temperatures were almost the same and reached an equilibrium state. After 90 min, the drying curves were completely reversed and were inversely proportional to temperature.

### Changes of flavonoids and astragaloside IV during thermal drying

**Fig 2:**
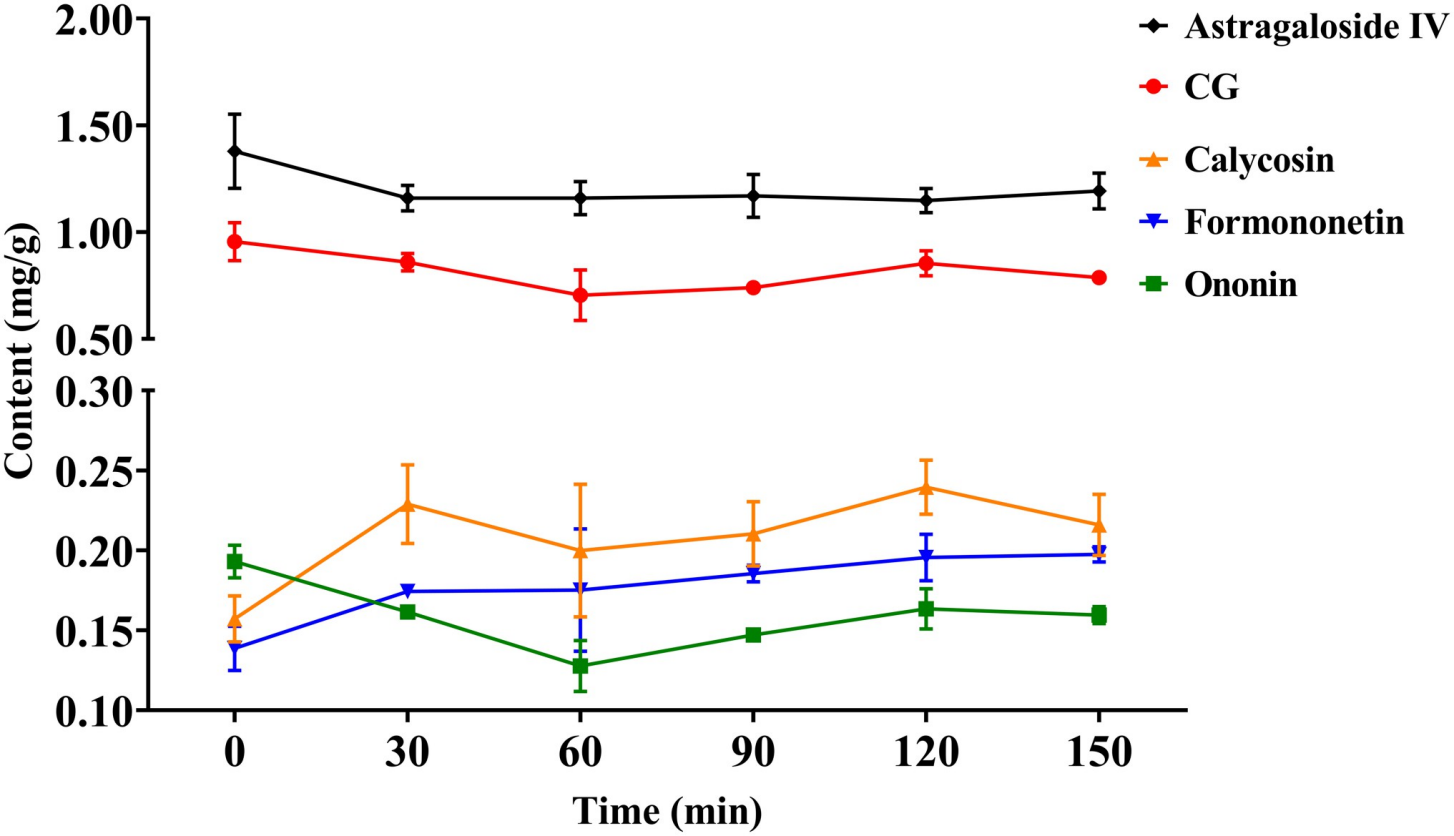
Contents of CG, calycosin, ononin, formononetin and astragaloside IV in AR pieces during drying at 80°C. Data are represented as the mean ± standard deviation of three independent experiments.

The contents of calycosin-7-*O*-*β*-*D*-glucopyranoside (CG), calycosin, ononin, formononetin and astragaloside IV, the four major flavonoids and one major saponin of AR, were measured. The total flavonoid content was determined by summing the content of each of the four flavonoids. The results are shown in Table 1. The content of CG was the highest after drying at 80°C, and the lowest at 70°C. As the drying temperature was increased, the total flavonoid and astragaloside IV content gradually increased, but there was no significant difference among three temperatures. The content of astragaloside IV ranged from 1.32 to 1.67 mg/g, with the highest content at 90°C, and the lowest at 70°C, this may be due to the conversion of other saponins to astragaloside IV [26]. At present, CG and astragaloside IV are used as index for the evaluation of AR quality in the Chinese Pharmacopoeia (2020 edition). By considering the contents of CG and astragaloside IV, it was concluded that 80 or 90°C was the optimal drying temperature in this work. However, after drying at 90°C, the color of the AR pieces deepened, along with a burnt smell, which did not meet the Chinese Pharmacopoeia requirements. So we chose 80°C as the best drying condition, and follow-up tests will also be conducted based on 80°C. As shown in the Fig 2, the contents of the four flavonoids and astragaloside IV in AR pieces dried at 80°C for different times did not change significantly, indicating that these components could exist stably in the AR pieces under this condition.

**Table 1:** Effects of different drying temperature on the contents of CG, ononin, calycosin, formononetin and astragaloside IV in AR pieces.

Drying temperature (°C)	Content (mg/g)					
	CG	ononin	calycosin	formononetin	total flavonoid	astragaloside IV
70	0.65 ± 0.03^b^	0.14 ± 0.02^a^	0.20 ± 0.01^b^	0.28 ± 0.01^a^	1.27 ± 0.05^a^	1.32 ± 0.02^b^
80	0.84 ± 0.09^a^	0.17 ± 0.01^a^	0.21 ± 0.01^b^	0.20 ± 0.01^b^	1.41 ± 0.11^a^	1.56 ± 0.13^a^
90	0.77 ± 0.10^ab^	0.15 ± 0.01^a^	0.26 ± 0.03^a^	0.27 ± 0.02^a^	1.45 ± 0.15^a^	1.67 ± 0.14^a^

Note: 1. Values in the table are means ± standard deviation; 2. a,b: values with the same superscripts are not significantly different (*P* > 0.05), and those with different superscripts are significantly different (*P* < 0.05).

### LF-NMR moisture analysis

**Fig 3:**
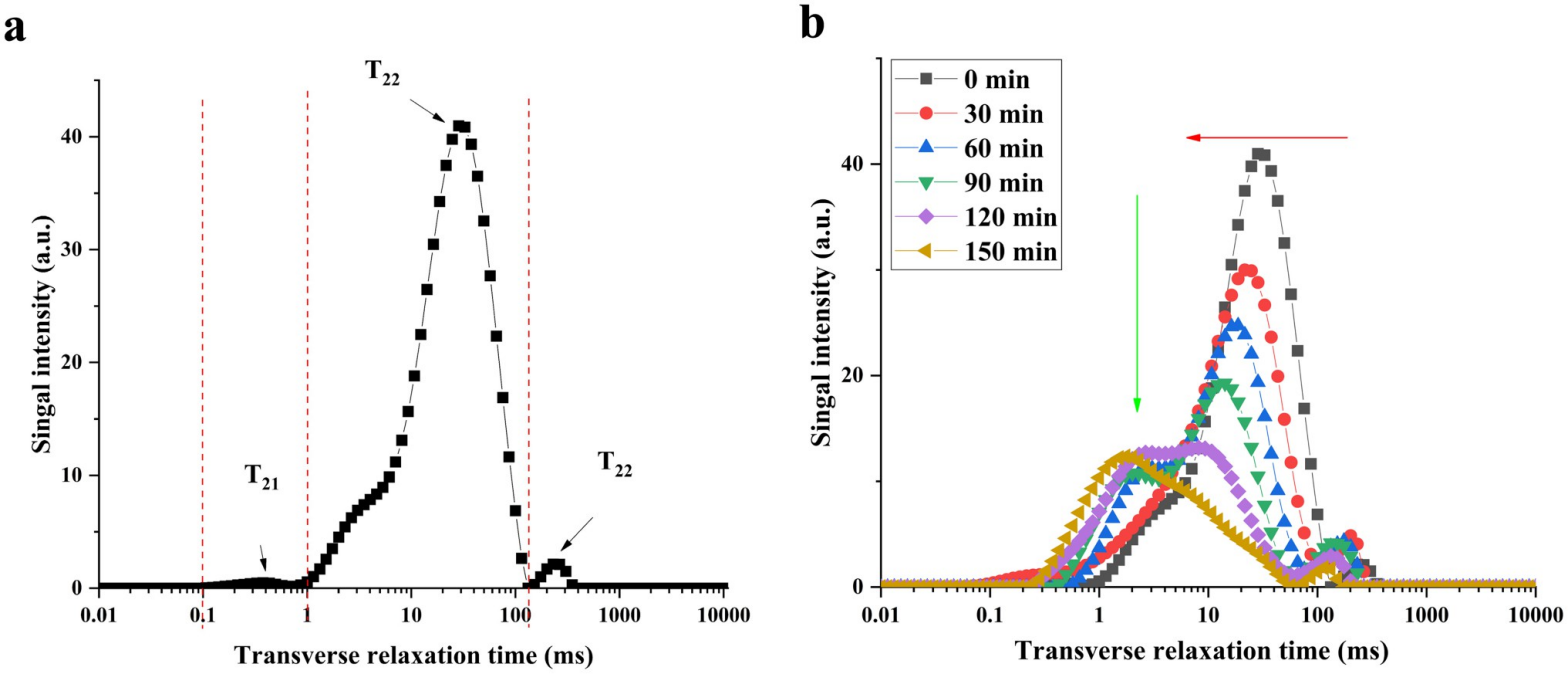
Transverse relaxation time (T_2_) curves for AR pieces during drying. (a): Sample not dried. (b): Samples at different drying time.

The T_2_ relaxation curves (Fig 3a) showed that AR pieces were mainly divided into two water groups during drying, and their relaxation times were different; the relaxation time between 0.01–10 ms was defined as T_21_, and 10–1000 ms was defined as T_22_, which corresponded to bound and free water, respectively. Since the water contents of the samples at different time were different and the relaxation intensity was related to the water content, it was necessary to normalize the quality of the samples. The results of bound water (A_21_), free water (A_22_) and total water (A_Total_) in the samples after normalization are shown in Table 2. The change trends of bound water, free water and total water values were completely opposite: during the drying treatment of bound water, both peak area and content showed an upward trend, but the values of free water and total water volume showed a downward trend. For moistened AR pieces, A_22_ and A_21_ accounted for 99.38% and 0.62% of the total water content, respectively. However, for thoroughly dried AR pieces, A_22_ and A_21_ accounted for 15.49% and 84.51% of the total water content, respectively. That is, the content of water present in the tissue spaces decreased during all drying processes, but the water content that binds to other substances increased (Fig 3b).

**Table 2:** Changes in signals per unit mass of AR pieces during hot-air drying at 80°C.

Drying time (min)	T_21_ (ms)	T_22_ (ms)	A_21_ (g^-1^)	A_22_ (g^-1^)	A_Total_ (g^-1^)	A_21_ (%)	A_22_ (%)
0	0.48± 0.16^d^	26.31± 3.40^a^	3.93± 0.83^e^	633.23± 24.09^a^	637.15± 23.55^a^	0.62	99.38
30	1.68± 0.12^c^	21.43± 1.71^b^	22.59± 2.24^de^	545.90± 20.09^b^	568.48± 23.12^b^	3.79	96.03
60	0.86± 0.17^d^	18.59± 0.37^b^	55.49± 3.68^d^	433.38± 12.39^c^	488.87± 15.12^c^	11.35	88.65
90	2.25± 0.25^ab^	14.93± 0.84^c^	104.52± 13.12^c^	253.76± 22.47^d^	358.28± 32.91^d^	29.14	70.86
120	2.50± 0.29^a^	19.30± 1.04^b^	138.55± 13.95^b^	201.60± 7.94^e^	340.15± 18.48^de^	40.68	59.32
150	2.00± 0.27^bc^	22.43± 1.38^b^	251.06± 41.26^a^	45.24± 1.98^f^	296.30± 40.73^e^	84.51	15.49

Note: 1. Values in the table are means ± standard deviation; 2. a–f: values with the same superscripts are not significantly different (*P* > 0.05), and those with different superscripts are significantly different (*P* < 0.05).

### MRI water migration analysis

**Fig 4:**
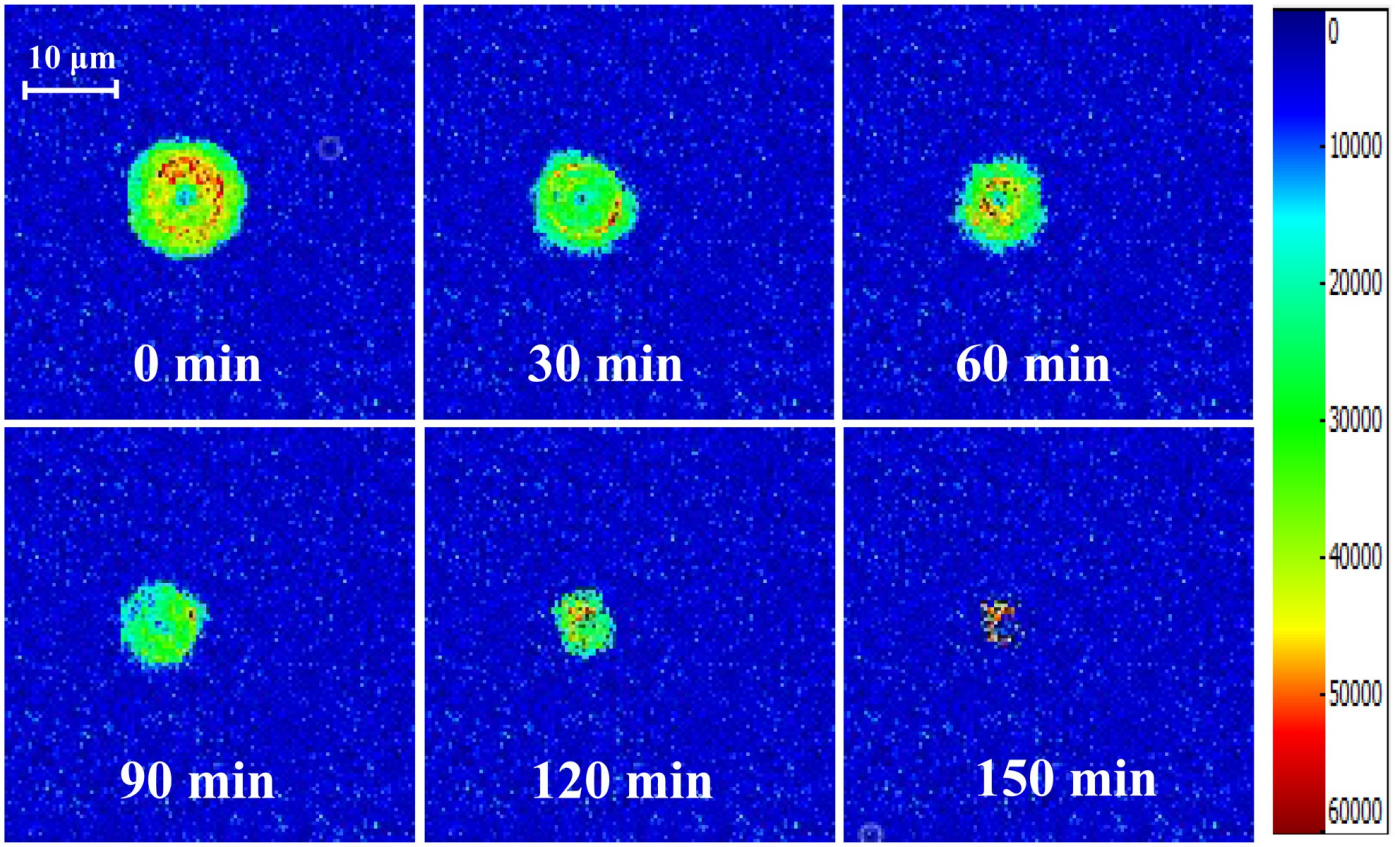
T_2_-weighted MRI images of AR pieces drying at 80°C.

Fig 4 shows T_2_-weighted MRI images of AR pieces dried at 80°C. The red area represents high moisture content, the yellow area represents low moisture content, and the green area represents the least moisture content. The legend on the right side indicates that the H^+^ proton density changes from high to low, and the color changes from red to blue. The changes in H^+^ protons echo the changes in the T_2_ inversion spectrum with drying time above. As drying proceeded, the area of the MRI images of the AR pieces gradually shrunk; the H^+^ proton density gradually decreased, and the signal color eventually changed to light blue, indicating that the water content of AR pieces gradually decreased. The MRI images showed that the relaxation signals for H^+^ protons outside the AR pieces weakened initially, indicating that free water was mostly distributed outside the AR pieces; in contrast, bound water was primarily distributed inside the AR pieces.

### Texture changes

Table 3 shows the textural parameters of the hardness, adhesion, springiness, and fracturability properties of AR pieces during the drying process. With the extension of drying time, the hardness, adhesion and fracturability properties increased significantly, especially in the final drying stage. At the end of drying, the hardness value increased from 686.33 g to 2656.67 g, the adhesion value increased from 160.00 g to 1163.67 g, and the fracturability value increased from 189.67 g to 520.00 g.

**Table 3:** Changes in textural parameters of AR pieces at different drying times.

Drying time (min)	Hardness (g)	Adhesion (g)	Springiness	Fracturability (g)
0	686.33 ± 23.18^f^	160.00 ± 5.57^e^	0.06 ± 0.02^ab^	189.67 ± 69.29^b^
30	806.00 ± 37.03^e^	197.67 ± 80.06^e^	0.06 ± 0.02^ab^	283.67 ± 60.38^ab^
60	1094.00 ± 60.56^d^	303.00 ± 51.12^d^	0.06 ± 0.01^a^	325.00 ± 132.53^ab^
90	1490.00 ± 67.62^c^	501.00 ± 28.05^c^	0.06 ± 0.01^a^	486.33 ± 92.80^ab^
120	2016.67 ± 56.62^b^	844.67 ± 51.39^b^	0.03 ± 0.01^b^	511.00 ± 290.29^a^
150	2656.67 ± 43.84^a^	1163.67 ± 29.91^a^	0.06 ± 0.01^ab^	520.00 ± 128.85^a^

Note: 1. Values in the table are means ± standard deviation; 2. a–f: values with the same superscripts are not significantly different (*P* > 0.05), and those with different superscripts are significantly different (*P* < 0.05).

**Fig 5:**
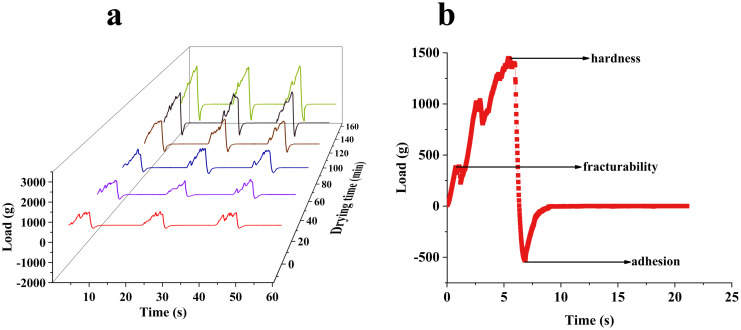
Load curves for AR pieces during drying. (a): Samples at different drying times. (b): One complete test.

**Fig 6:**
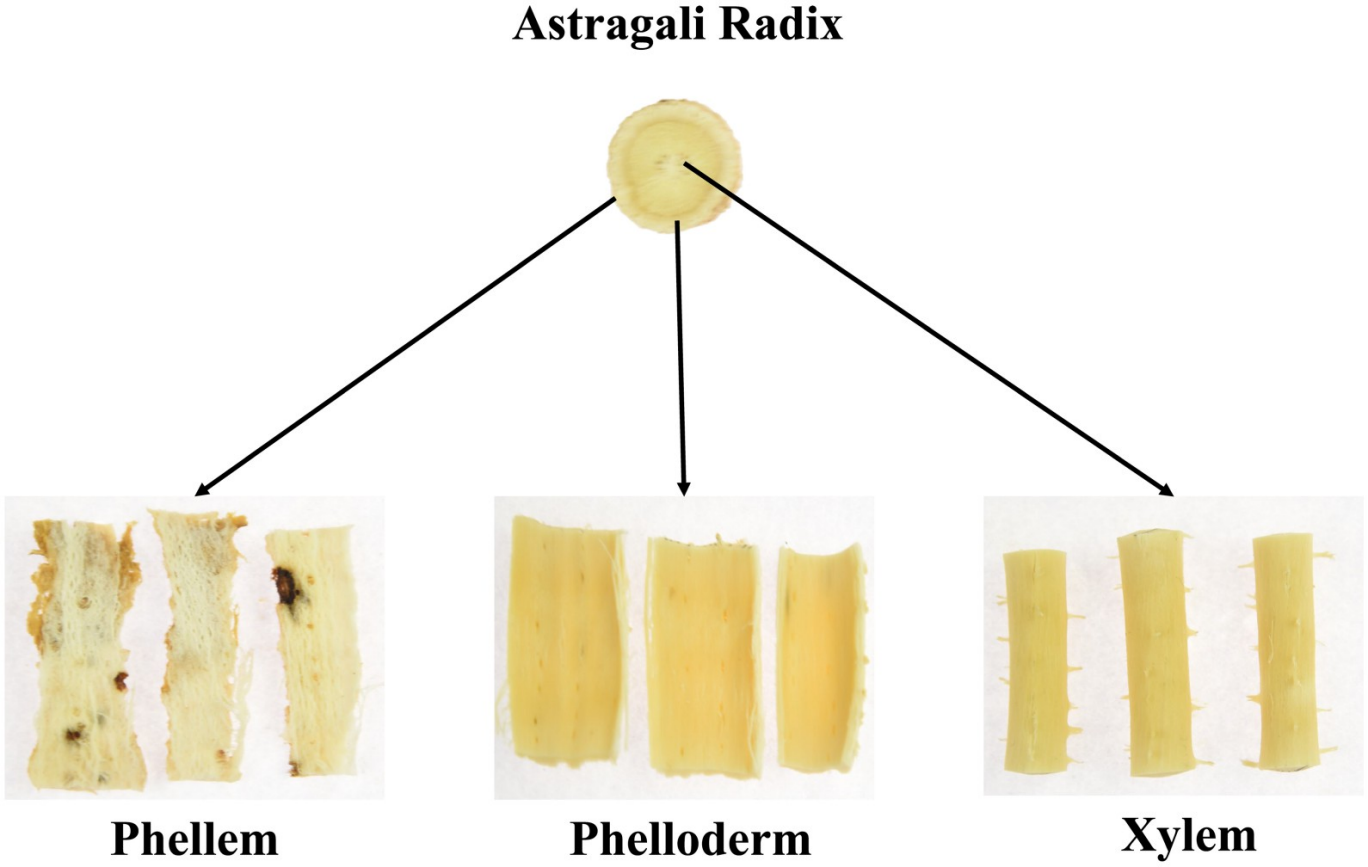
Anatomy of Astragali Radix.

Fig 5 contains the loading curves of the AR pieces during the drying process. Fig 5a clearly shows that the hardness of AR increased significantly during drying. From 0 min to 60 min, the hardness did not change obviously, but the value increased significantly from 60 min to 150 min. Fig 5b shows a complete test. From the figure, we know key information such as hardness, fracturability, and adhesion. The measurement curve appeared in the region of negative vertical coordinates in Fig 5b resulting from AR squeezing the puncture needle during the return stroke after completion of the puncture. At the same time, the curve was not completely smooth (Fig 6). Based on its anatomy, Astragali Radix can be roughly divided into three parts: phellem, phelloderm and xylem. Due to the different functions of the three tissue structures, the types and arrangements of the cells within them are different, resulting in different hardness values and ultimately in an unsmooth hardness curve, this may lead to measurement errors.

**Fig 7:**
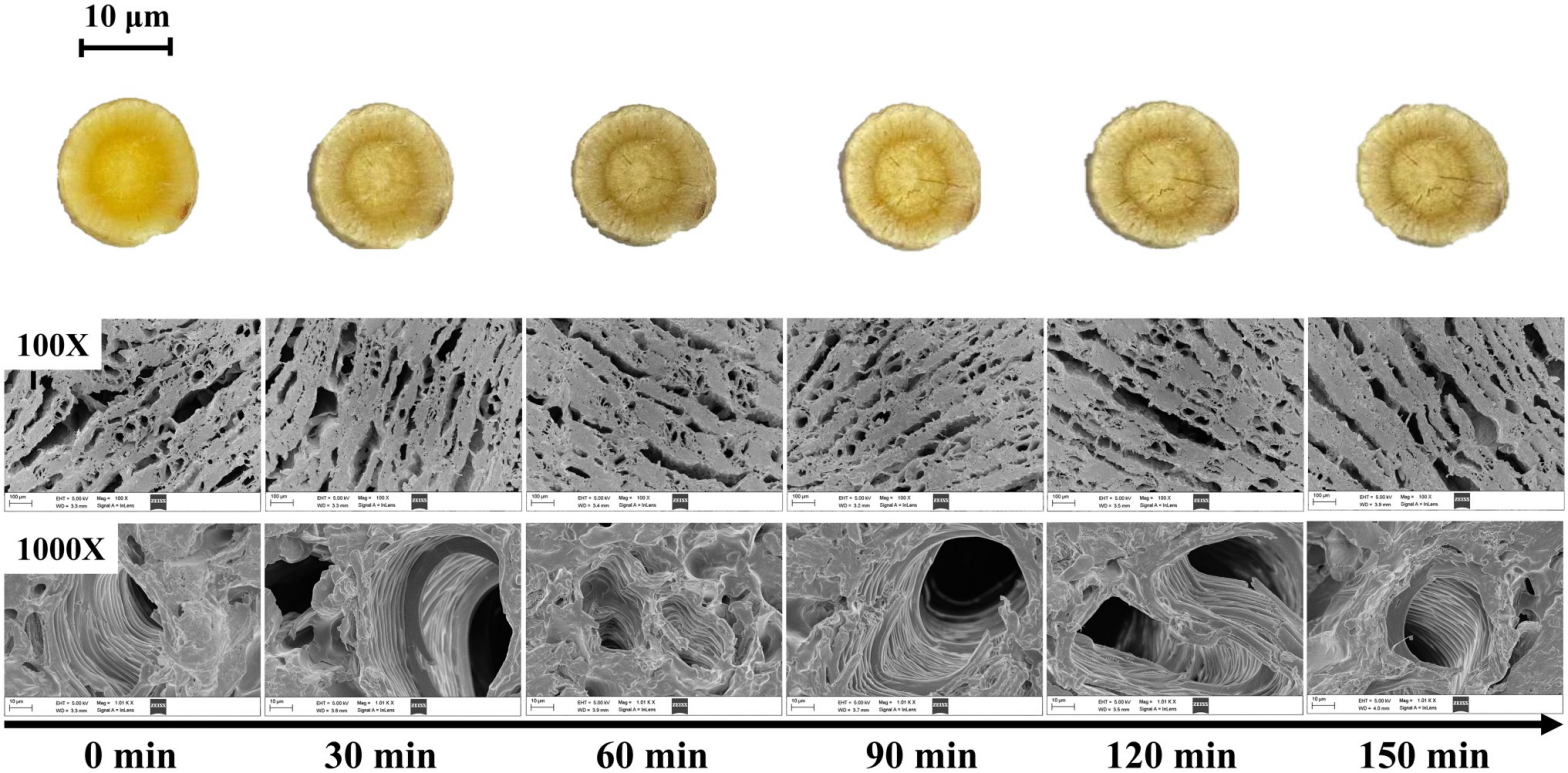
Scanning electron micrographs of 100X (first) and 1000X (second) for AR pieces at different drying times.

Scanning electron microscopy confirmed this idea from a microscopic point of view. As shown in Fig 7, the microstructure of Astragalus *membranaceus* did not change much during the drying process, indicating that Astragalus *membranaceus* contained more fibrous components and dense cellular mass. However, plants with a predominantly natural component of fiber tend to maintain the integrity of their voids and channels when they are heated or otherwise processed [27].

### Correlation analysis of moisture content, LF-NMR data and textural parameters

To determine the relationship among moisture content, low field NMR data and textural parameters, we used Pearson correlation analysis to analyze moisture content, five low field NMR parameters (T_21_, T_22_, A_21_, A_22_ and A_Total_) and four texture data (hardness, adhesion, springiness, and fracturability). From Table 4, it is known that the moisture content showed a significant negative correlation with the values of T_21_ and A_21_ but a strong positive correlation with the values of T_22_, A_22_ and A_Total_. Positive correlation coefficients *r* values up to 0.915, while negative correlation coefficients up to − 0.830. This indicated that the water content of the herbs gradually decreased with the extension of drying time, resulting in a tendency for the values of T_21_ and A_21_ to increase, while the values of T_22_, A_22_ and A_Total_ to decrease. LF-NMR data were related to texture analyzer parameters. Among them, T_21_ and A_21_ had some certain positive correlation, while the remaining data showed a negative correlation with hardness, adhesion and fracturability. Surprisingly, the springiness was independent of most parameters (including MC), which may be related to the fact that AR pieces are inflexible.

**Table 4:** Correlation coefficients for moisture content (MC), LF-NMR and texture parameters.

	**MC (%)**	**T**_**21**_ **(ms)**	**T**_**22**_ **(ms)**	**A**_**21**_ **(g**^**−1**^**)**	**A**_**22**_ **(g**^**−1**^**)**	**A**_**Total**_ **(g**^**−1**^**)**	**Hardness (g)**	**Adhesion (g)**	**Springiness (%)**	**Fracturability (g)**
MC (%)	1	− 0.750**	0.622**	− 0.821**	0.915**	0.901**	− 0.695**	− 0.650**	0.310	− 0.612**
T_21_ (ms)		1	− 0.450*	0.616**	− 0.672**	− 0.655**	0.454*	0.445*	− 0.355	0.348
T_22_ (ms)			1	− 0.203	0.456*	0.556**	− 0.389	− 0.399	0.189	− 0.530**
A_21_ (g^−1^)				1	− 0.912**	− 0.804**	0.899**	0.659**	− 0.258	0.516**
A_22_ (g^−1^)					1	0.977**	− 0.883**	− 0.843**	0.408*	− 0.750**
A_Total_ (g^−1^)						1	− 0.909**	− 0.880**	0.458*	− 0.820**
Hardness (g)							1	0.984**	− 0.518**	0.830**
Adhesion (g)								1	− 0.543**	0.840**
Springiness (%)									1	− 0.405*
Fracturability (g)										1

**P<0.01, significant at the 0.01 level.

*P<0.05, significant at the 0.05 level.

## Discussion

Various factors, such as drying temperature, product thickness, drying method and drying time, will have a profound impact on the drying characteristics of the product [28]. Irrespective of the drying temperature, the drying process of AR pieces mainly stayed in the decreasing rate stage, indicating that diffusion was limited by internal moisture transfer [29], since drying efficiency depends on the difficulty of capillary diffusion and the nature of the material [30]. These results are consistent with the findings for other biomaterials [31–33]. The whole process of drying AR pieces was mainly characterized by increasing speed and decreasing period, and there was almost no continuous period, which was consistent with many previous studies [34, 35].

One of the important indicators to evaluate the quality of Chinese herbal medicines is the chemical composition [36]. The metabolic efficiencies for digestion of flavonoid glycosides and flavonoid aglycones in the human small intestine are different [37], so monitoring the changes in index components during drying of a TCM is of great significance in controlling the quality of TCM decoction pieces. The results obtained in the present study showed that high-temperature drying treatments would not led to significant dynamic changes in flavonoids and astragaloside IV contents in AR pieces.

Monitoring of T_2_ values during drying of AR pieces using LF-NMR to investigate their internal moisture status during drying [20]. The longer the T_2_ relaxation time, the weaker or freer the hydrogen binding. Water in the cell wall produces T_21_ relaxation, characterized by water molecules (hydrated unimolecular membranes) bound to strong hydrogen bonds. In contrast, water in the extracellular and cytoplasmic space produces T_22_ relaxation, where water molecules are tightly bound to a single layer (multilayer water) [38].

With the drying proceeded, the T_2_ relaxation time curves gradually moved to the left, the area gradually decreased, which means that the water became less fluid and underwent a certain degree of quantitative change. The values of A_22_ were greater than those of A_21_ at the beginning of drying, but A_21_ was much larger than A_22_ in the end. This suggested that most of the water lost in the drying process of AR pieces came from free water rather than bound water. The *r* values of the correlation analysis showed that the dynamic process of water loss can be monitored by fast and nondestructive LF-NMR technology, and the abstract process can be concretized.

MRI is a new technique that can provide real-time information on the distribution and mobility of water molecules. It is commonly used for quantitative detection due to its noninvasive and fast characteristics, and it provides information on water mobility by detecting the spin-spin relaxation time (T_2_) of protons. However, oils and fats are also rich in protons, which results in MRI not being qualitatively accurate [39]. Bright MRI images reflect stronger response values and higher H^+^ proton density. In contrast, the lower H^+^ proton density reflected in the image is a darker color [40]. With the decreased MRI image area of AR samples, the red part converted to green and the green part transformed to blue during the extension of drying time. It can be seen from the MRI pseudo color map of AR pieces after drying for 0 min that the H^+^ proton density inside the pieces was greater than that outside, indicating that there was more free water inside and more bound water outside. Drying time was directly related to moisture reduction, and the signal intensity of T_2_-weighted images showed significant changes in AR piece moisture distribution during drying. The mobility of moisture inside the samples was lower than that of moisture on the surface. In other words, the areas close to the surface and exposed to the drying environment were prone to moisture loss and reduced H^+^ signal intensity. This indicated that hot air drying was a drying process from the outside to the inside, while microwave freeze drying was a uniform drying process with consistent drying inside and outside [41]. A small part of the red color in the MRI image of dried AR pieces may be due to the texture of the medicinal material, which made it difficult to remove the internal water, leaving part of the free water.

Texture is very important for the quality control of Chinese herb medicines, hardness is one of the most important texture factors. After drying, the interiors of the AR pieces were denser than those of the moistened pieces. Natural plant fibers are natural polymers with high strength and high stiffness. It is the main framework of long fiber cells with diverse forms and complex structures. The cells that act as supporting tissues in plant stems and roots are fibroblasts that die after maturation [42]. Due to the highly fibrous nature of the samples, adhesion gradually increased. The moistened AR samples were less brittle and more easily bent than the dried samples, which may be a macro manifestation of increased fracturability. However, the springiness did not change significantly in our study. The drying process of Chinese herbal medicine results in a large amount of water loss, causing AR cells or tissue structures to change, especially after the loss of water sample volume reduction, surface wrinkling and fiber arrangement to be denser, resulting in increased hardness and fracturability. Nevertheless, due to the morphological differences of traditional Chinese medicines and the uneven texture of each parts, the sensory evaluation of texture instrument was difficult to be completely unified.

The correlation among moisture content, five LF-NMR parameters and four texture parameters was obtained through correlation analysis. Therefore, the water content, texture change and water distribution can be predicted using the total signal amplitude. It has also been demonstrated that LF-NMR/MRI and texture analyzers are convenient technologies for clarifying the drying mechanism during the process.

## Conclusion

In this study, three equations and four instruments were combined to study the drying mechanism of AR pieces and achieved quantitative and visual results; there was a correlation between these parameters, which made it possible to guarantee the quality of prepared pieces after the drying process or to optimize the process parameters. Accordingly, it can easily be extended to other traditional Chinese medicinal materials for radixes or rhizomes. Future research should be devoted to the establishment of different drying models and the use of the internet to combine these models with LF-NMR/MRI to achieve intelligent management, saving human resources and thus ensuring controllable production processes.

## Supporting information

S1 Data (XLSX)
